# Protocol for the SEED-trial: Supported Employment and preventing Early Disability

**DOI:** 10.1186/s12889-016-3280-x

**Published:** 2016-07-15

**Authors:** Vigdis Sveinsdottir, Torill Helene Tveito, Gary R. Bond, Astrid Louise Grasdal, Stein Atle Lie, Silje Endresen Reme

**Affiliations:** Uni Research Health, Uni Research, POB 7810, 5020 Bergen, Norway; Dartmouth Psychiatric Research Center, Rivermill Commercial Center, 85 Mechanic St., Suite B4-1, Lebanon, NH 03766 USA; Department of Economics, University of Bergen, POB 7802, 5020 Bergen, Norway; Department of Clinical Dentistry, University of Bergen, POB 7804, 5020 Bergen, Norway; Department of Psychology, University of Oslo, POB 1094, 0317 Oslo, Norway

**Keywords:** Disability, Employment, Individual Placement and Support, Randomized Controlled Trial, Supported Employment, Vocational Rehabilitation, Unemployment, Work Disability, Youth

## Abstract

**Background:**

Early withdrawal or exclusion from the labor market leads to significant personal and societal costs. In Norway, the increasing numbers of young adults receiving disability pension is a growing problem. While a large body of research demonstrates positive effects of Supported Employment (SE) in patients with severe mental illness, no studies have yet investigated the effectiveness of SE in young adults with a range of social and health conditions who are receiving benefits.

**Methods/design:**

The SEED-trial is a randomized controlled trial (RCT) comparing traditional vocational rehabilitation (TVR) to SE in 124 unemployed individuals between the ages of 18-29 who are receiving benefits due to various social- or health-related problems. The primary outcome is labor market participation during the first year after enrollment. Secondary outcomes include physical and mental health, health behaviors, and well-being, collected at baseline, 6, and 12 months. A cost-benefit analysis will also be conducted.

**Discussion:**

The SEED-trial is the first RCT to compare SE to TVR in this important and vulnerable group, at risk of being excluded from working life at an early age.

**Trial registration:**

Clinicaltrials.gov, registration number NCT02375074. Registered on December 3rd 2014

## Background

### Early disability

The number of young adults receiving permanent disability pension in Norway has recently accelerated. While the overall percentage of disability pensioners in the population has remained steady for the last decade, there has been an increase in the proportion of young disability pensioners (between 18 and 29 years old) and a continuous decline in older disability pensioners throughout the same period [1, 2]. From 2006 to 2015 there was an increase of 77 % in young disability pensioners [3] while the population in the same age group increased by 23 % [4], making the development evident even when population growth is accounted for. Additionally, during the same period, an increased rate of labor immigration (mainly young males) has inflated the number of working young adults, which may have suppressed the percentage growth and led to an underestimation of the development [1].

Musculoskeletal and common mental disorders account for about 2/3 of sickness benefits and disability pensions issued in Norway [5, 6], but within the subgroup of disability pensioners aged 18–29, 59 % are receiving disability pensions due to mental illness and behavior disorders alone [7]. This type of early withdrawal or exclusion from the labor market leads to vast personal and societal costs, especially when seen in context with the aging of the Norwegian population causing a disparity between the supply of available workforce and the need of work capacity [8]. Furthermore, the importance of work for health and well-being is well-documented [4–6], and evidence shows that unemployment is not only caused by mental health problems, but also causes them [7, 8].

Long-term sickness absence is a risk factor for unemployment and permanent disability that goes beyond the effect of health status, suggesting that long-lasting absence may itself initiate a process of marginalization from the labor market [9]. Few recipients of long-term sickness benefits return to working life, seemingly due to mechanisms other than age, diagnosis, gender and public health [10]. This may be particularly relevant for young people in need of special assistance to obtain work, who are at risk of being excluded from working life before having had the chance to establish themselves on the labor market. Previous studies document that a small percentage of the population accounts for the majority of sickness absence, and that broad interventions targeting the workforce as a whole may not reach these small but high-risk groups [11]. Focusing on the group of young people who are receiving temporary benefits, but have not reached the point of more permanent disability pensions, thus appears to be a viable way to move forward.

### Perspectives in vocational rehabilitation

Vocational rehabilitation has traditionally been characterized by a *train-then-place principle*, involving prevocational training in sheltered environments before attempting to enter the open labor market [12]. In the train-then-place approach, clients try different forms of work adapted to their skills and challenges, while undergoing a stepwise process of targeted training to prepare them for competitive employment. Training is usually provided in group settings along with other workers with challenges or disabilities, and with close follow-up from an advisor. While the goal is to improve clients’ opportunities for obtaining work, the approach has been criticized for promoting dependency and demoralization [13], and for having a negative effect on different stakeholders expectations of the clients’ work ability and productivity [14].

In the 1980s, rehabilitation leaders in the U.S. introduced an approach based on the *place-then-train principle*, with a main goal of competitive employment and immediate work integration, without prevocational training [12]. This approach challenged common assumptions about people with serious disabilities being able to work only in workshops or other sheltered environments. Approaches within this perspective are known as Supported Employment (SE), and the evidence-based and manualized methodology of SE is called Individual Placement and Support (IPS). The model was originally developed for people with severe mental illness (SMI), and is supported by evidence from randomized controlled trials (RCT’s) in the US [15–24] as well as internationally [25–37], showing SE to be effective in this disability group on a range of vocational outcome measures. IPS involves individual support from a trained job specialist, incorporating eight evidence-based principles: focus on competitive employment in ordinary paid positions; rapid job search, starting the job search on average within one month after program entry; attention to the client’s choices and preferences; integrating work with mental health treatment; personalized benefits counseling; systematic job development; individualized long-term job support; and eligibility based on the client’s choice [38]. The latter involves a zero exclusion criteria, which states that everyone who has an expressed desire to work should have access to IPS services regardless of factors such as previous employment history, history of violent behavior, personal presentation, or substance abuse, and that the service does not screen for work readiness [39].

Evidence-based knowledge of the effectiveness of the services being offered through public agencies is of vital importance in future planning of vocational rehabilitation of young adults. Although there is a large and growing body of research demonstrating the effectiveness of the IPS approach in other populations, no studies have yet examined the effectiveness of IPS specifically for young adults at risk of becoming permanent disability pensioners.

## Methods/Design

The study is conducted by Uni Research Health, in collaboration with the Norwegian Labor and Welfare Administration (NAV).

### Aims and objectives

The aim of the project Supported Employment and preventing Early Disability (SEED-trial) is to compare two interventions to increase labor market participation in young people at risk of early work disability: Traditional Vocational Rehabilitation (TVR) versus Supported Employment (SE).

### Background measures

Each participant will be asked to complete questionnaires including background information on demographics and employment history.

### Outcome measures

#### Primary outcome: competitive employment

The primary outcome of the SEED-trial is *competitive employment at any time during the 12 months after enrollment in the study*. Competitive employment is here defined as working in a job on the competitive labor market, at usual wages, with regular supervision.

Additionally, success in employment will be defined using a range of standardized indicators of employment outcomes used in IPS studies [40], including rate of job acquisition, amount and duration of work, total wages, and number of days from enrollment in the study to employment. Information about receipt of social security benefits (sickness and disability benefits, unemployment, work assessment allowance), income, financial assistance, and educational activity (started or completed education), will also be collected.

We will use three sources of information for competitive employment: Survey data for hours worked and success in employment; register data from the NAV for receipt of social security benefits and income; and register data from Statistics Norway (SSB) for financial assistance and educational activity.

#### Secondary outcomes: self-reported health and well-being

Questionnaires distributed to all participants will further measure a range of secondary and non-vocational outcomes related to health and well-being, including interventions and treatment received for the last 6 months, experiences with bullying and violence, sleep problems, and the following variables:

- *Alcohol and drug abuse* will be measured using the 3-item Alcohol Use Disorders Identification Test (AUDIT-C) [41] screening for problem drinking, and the 11-item Drug Use Disorders Identification Test (DUDIT) [42] screening for drug-related problems and drug dependence.
- *Coping* will be measured using the 7-item Theoretically Originated Measure of the Cognitive Activation Theory of Stress (TOMCATS) [43], consisting of 3 subscales: coping (1 item), helplessness (3 items), and hopelessness (3 items).
- *Disability* will be measured using the 12-item version of the WHO Disability Assessment Schedule 2.0 (WHODAS 2.0), measuring functioning in 6 domains of life: cognition (2 items), mobility (2 items), self-care (2 items), getting along (2 items), life activities (2 items), and participation (2 items) [44].
- *Fatigue* will be measured using the 11-item Chalder Fatigue Questionnaire (CFQ) consisting of 2 subscales: physical fatigue (7 items) and mental fatigue (4 items) [45].
- *Illness perceptions* will be measured using the 9-item Brief Illness Perception Questionnaire (B-IPQ) [46], measuring 9 dimensions of illness perceptions: consequences (1 item), timeline (1 item), personal control (1 item), treatment control (1 item), identity (1 item), coherence (1 item), emotional representation (1 item), and concern (1 item), in addition to an open-ended item concerning causal factors (1 item).
- *Mental health* will be measured using the 25-item Hopkins Symptom Checklist (HSCL-25) [47], consisting of 2 subscales: anxiety symptoms (10 items) and depression symptoms (15 items).
- *Social support* will be measured using a revised 11-item version of the Social Support Inventory [48, 49] using 2 subscales as suggested by Øyeflaten et al. [50]: directive social support (4 items) and nondirective social support (7 items).
- *Subjective health complaints* will be measured using the 29-item Subjective Health Complaints Inventory (SHC), consisting of 5 subscales: musculoskeletal pain (8 items), pseudoneurology (7 items), gastrointestinal problems (7 items), allergy (5 items), and flu (2 items) [51].
- *Quality of life* will be measured using the 5-item EuroQol questionnaire (EQ-5D) including a visual analogue scale (EQ-VAS) [52].

### Participants and randomization

#### Inclusion and exclusion

Eligible participants will consist of unemployed individuals aged < 30 years old, who are receiving temporary benefits due to various social- or health-related problems. Attending employment services overseen by the NAV is a requirement for recipients of these benefits, and we will invite all those who are intended for the specific traditional employment service called “traineeship in a sheltered business”. The only additional exclusion criteria are that participants must have an expressed desire to work and sufficient language skills to answer questionnaires in Norwegian.

#### Recruitment and randomization

Nine local NAV-offices throughout the Hordaland County are involved in the project. Caseworkers at each office will refer all eligible participants to general information meetings organized by researchers at Uni Research Health in collaboration with NAV. The meetings include detailed information about the project and invitation to participate in the study. Interested individuals will be asked to read and give informed consent, and researchers will record their personal information (name, contact details, and national identification number) and provide each participant with an ID-number on the spot. ID-numbers will be randomized at Uni Research Health after the meetings, using premade computer-generated lists with a 1:1 randomization ratio. Information about randomization outcome will be communicated by e-mail or telephone to the relevant caseworker at NAV, who contacts their client and the relevant vocational rehabilitation organization.

### Data collection and data management

Survey data will be collected at baseline, 6 and 12 months. Baseline questionnaires will be administered at the information meetings, and participants complete their information electronically on iPads with secure software (Qualtrics®), or in paper format if preferred. Follow-up questionnaires will be administered electronically to participants providing their e-mail address at baseline, or in paper format via regular mail.

Data collected using iPads will automatically be electronically transferred to and stored in a secure online database. Data collected in paper form will be entered manually by the data manager at Uni Research Health and sent to the same database, after which the original questionnaires will be stored in a locked filing cabinet.

Register data will be collected retrospectively for 3 years before baseline, and for a 5-year period after enrollment date. The information will be de-identified and merged with survey data, while the identifier is secured in a locked and fireproof safe.

### Study design

**Fig. 1:**
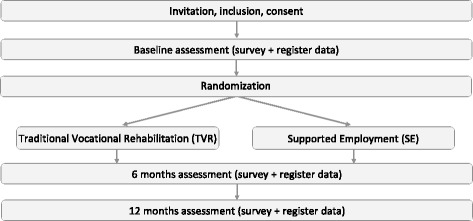
Study design

The SEED-trial is designed as a randomized controlled trial (RCT), and participants are randomly assigned to 1 of 2 interventions (Fig. 1).

#### Interventions

Both interventions in this trial are offered by VR organizations overseen by the NAV, and are part of the various employment schemes offered to people on temporary benefits in Norway. Participants continue to receive temporary benefits while attending the services, which are normally offered for up to two or three years, depending on the specific intervention and individual needs.

##### Traditional Vocational Rehabilitation (TVR)

The first group will be referred to a TVR organization, where they will receive a traineeship in a sheltered business with follow-up from trained advisors and department supervisors.

This intervention is *service as usual* for the study participants, and is currently offered to clients who are considered by their caseworker to have need for special assistance to obtain work. The specific approach falls within the *train-then-place principle*, and participants receive preparatory work training in a sheltered environment before pursuing employment. The goal of the intervention is to improve the participant’s work skills and opportunities for entering the labor market, and includes follow-up geared towards finding a job. The traineeships are offered by various sheltered businesses in the area with a range of departments including canteens and catering, car repair, day-care services, upholstery and interior decoration, transport, laundry services, welding, and warehouse handling. The distribution of participants to the various departments will follow usual practice, and is conducted at NAV based on the individual caseworker’s description of the client’s interests and goals, as well as availability and waiting-lists.

##### Supported Employment (SE)

The second group will be referred to the vocational rehabilitation organization Fretex Vest-Norge, where they will receive SE by trained job specialists following the evidence-based principles of IPS SE.

The intervention is based on the *place-then-train principle*, aiming to help people with health problems or other challenges participate in the competitive labor market, without the use of prevocational training, stepwise and sheltered approaches, or make-work jobs. It aims to find a good job match for the individual followed by on-the-job support after employment, and is based on a belief that anyone who wants to work can hold a job in the normal labor market as long as it is the right job and work environment for that individual.

#### Adaptions to the IPS SE model

As the manualized intervention of IPS SE was originally developed for patients with SMI, job specialists will need to make some adjustments to the services offered based on the individual participant’s challenges. One necessary adjustment concerns the principle of integrating employment services with mental health treatment, as that will not be applicable for participants that do not suffer from mental illness. In cases where participants are receiving treatment for other health problems, job specialists will establish contact with their respective health practitioners instead.

The implementation will be led by an experienced IPS trainer, who will also be in charge of the fidelity reviews, using the IPS fidelity scale [53], which is a standardized and validated scale for measuring adherence to the IPS model [54]. Fidelity reviews will be conducted using document review, calendar review, observations, and interviews of the different stakeholders, in order to determine to what degree the SE intervention fulfills the criteria for IPS SE. These evaluations will be used for quality improvement of services throughout the study period, aiming to adhere to the manualized and evidence-based treatment in spite of the necessary adaptions.

#### Sample size calculation

Our estimates of sample size are based on international input-data from previous IPS-studies where a mean competitive employment rate of 61 % has been found for IPS and 23 % for controls [55]. If we use 61 and 23 % as possible employment rates, we will need 31 participants in each group in order to obtain a statistical significant difference (with a 5 % significance level and power of 90 %). In order to enable stratified analyses to investigate treatment effects for sub-groups (e.g. for gender), we aim at including a total of 124 participants. The inclusion period will last for up to 2 years and close when the targeted number of participants has been reached.

#### Statistical analyses

Assessment of treatment effects will be analyzed using standard statistical methods, including t-tests for continuous data and chi-square tests for categorical data. Logistic regression will be performed to study potential moderators of treatment effects. For repeated measures over time (e.g. for sick leave), the statistical analyses may be extended to generalized estimation equations (GEE), to account for correlated data. All analyses will follow the intention to treat principle.

#### Cost-benefit analysis

Economic returns will be calculated based on treatment effects obtained from the statistical analyses, and will be evaluated using a standard cost benefit formula [56–58], as used by Hagen et al. [59].

Benefit will be measured in terms of increases in the net present value of production, as indicated by an increase in labor market participation. This is calculated as the product of the treatment effect, i.e. the increase in labor market participation and the productivity gains for the society when a person is employed as opposed to receiving social security benefits. Cost of the intervention is measured by treatment cost and costs related to follow-up outside the intervention in the different treatment groups. Health care utilization will be measured using survey data from the participants providing information about health and use of health services.

## Discussion

The SEED-trial will provide new knowledge about the effect of TVR versus SE in increasing labor market participation among young unemployed with various social- and health related problems. It will be the first RCT to look at SE for this important and vulnerable group at risk of being excluded from the labor market even before they have had the chance to establish themselves on the labor market.

## Abbreviations

APS, traineeship in a sheltered business; IPS, individual placement and support; NAV, Norwegian labor and welfare administration; RCN, Research Council of Norway; RCT, randomized controlled trial; REC, Norwegian Regional Committees for Medical and Health Research Ethics; SE, Supported Employment; SSB, Statistics Norway; TMG, trial management group; TSC, trial steering committee; TVR, Traditional Vocational Rehabilitation
